# Vitamin D and Metabolic Syndrome: Molecular Mechanisms and Clinical Implications: A Narrative Review

**DOI:** 10.3390/ijms27167101

**Published:** 2026-08-07

**Authors:** Héctor Fuentes-Barría, Raúl Aguilera-Eguía, Miguel Alarcón-Rivera, Cherie Flores-Fernández

**Affiliations:** 1 Centro de Investigación en Medicina de Altura (CEIMA), Universidad Arturo Prat, Iquique 1110939, Chile; 2 Departamento de Salud Pública, Facultad de Medicina, Universidad Católica de la Santísima Concepción, Concepción 3349001, Chile; raguilerae@ucsc.cl; 3 Dirección de investigación, Universidad Bernardo O’Higgins, Santiago 8370993, Chile; m.alarconrivera@outlook.cl; 4 Departamento de Gestión de la Información, Universidad Tecnológica Metropolitana, Santiago 7550000, Chile; cflores@utem.cl

**Keywords:** vitamin D, metabolic syndrome, molecular biology, treatment outcome

## Abstract

Metabolic syndrome (MetS) is a complex multisystem disorder characterized by insulin resistance, central obesity, dyslipidemia, hypertension, and chronic low-grade inflammation, all of which substantially increase the risk of type 2 diabetes mellitus and cardiovascular disease. The aim of this narrative review is to examine the role of vitamin D in the pathophysiology of MetS from a multisystem perspective. Specifically, it synthesizes current evidence on the molecular mechanisms through which vitamin D may influence inter-organ communication, insulin resistance, adipose tissue dysfunction, hepatic metabolism, skeletal muscle function, chronic inflammation, oxidative stress, and mitochondrial homeostasis, highlighting its potential contribution to the prevention and management of MetS. Current evidence indicates that MetS should not be regarded merely as a cluster of isolated metabolic abnormalities but rather as a disorder characterized by disrupted molecular signaling and impaired communication among metabolically active organs. In this context, experimental and preclinical evidence suggests that vitamin D, through activation of the vitamin D receptor (VDR), modulates key signaling pathways, including AMP-activated protein kinase (AMPK), the mechanistic target of rapamycin (mTOR), nuclear factor kappa B (NF-κB), and peroxisome proliferator-activated receptor gamma (PPAR-γ), thereby influencing insulin sensitivity, inflammation, oxidative stress, mitochondrial function, and metabolic homeostasis. Nevertheless, clinical evidence remains heterogeneous due, in part, to the lack of consensus regarding serum 25-hydroxyvitamin D thresholds for defining vitamin D status, as well as differences in baseline vitamin D concentrations, supplementation regimens, study populations, and methodological designs. Overall, the available evidence suggests that vitamin D should be considered an adjunct to lifestyle-based interventions rather than a stand-alone therapeutic strategy. Future research is warranted to clarify its clinical utility in the prevention and management of MetS.

## 1. Introduction

Metabolic syndrome (MetS) is one of the most prevalent metabolic disorders worldwide and represents a major public health challenge due to its strong association with type 2 diabetes mellitus (T2DM), cardiovascular disease (CVD), and premature mortality [1,2,3]. According to the International Diabetes Federation and other international organizations, the global prevalence of MetS has increased substantially over recent decades, affecting approximately one-quarter of the adult population worldwide, largely driven by obesity, sedentary lifestyles, and population aging [4,5]. Traditionally, MetS has been defined as a cluster of interrelated metabolic abnormalities, including central obesity, insulin resistance, dyslipidemia, hypertension, and impaired glucose metabolism [4,5,6]. However, growing evidence suggests that MetS should instead be understood as a complex multisystem disorder characterized by chronic metabolic dysfunction involving multiple organs and tissues [7,8,9,10,11].

In recent years, the pathophysiological understanding of MetS has evolved from a collection of isolated cardiometabolic risk factors to an integrative model emphasizing dynamic interactions among the adipose tissue, liver, skeletal muscle, pancreas, gut, immune system, and central nervous system [4,12,13,14]. These organs communicate through hormones, adipokines, cytokines, metabolites, and extracellular signaling molecules that collectively regulate energy balance, glucose homeostasis, lipid metabolism, and systemic inflammation [13,14]. Disruption of this inter-organ communication network contributes to insulin resistance, chronic low-grade inflammation, mitochondrial dysfunction, endothelial impairment, and progressive metabolic deterioration, all of which are hallmarks of MetS [15]. Despite this increasing recognition of MetS as a multisystem disorder, the mechanisms regulating the interaction between metabolic organs remain incompletely understood, particularly regarding how endocrine and molecular signals integrate tissue-specific responses during metabolic deterioration [1,2,3]. Therefore, identifying potential regulators of inter-organ communication represents an important unresolved question in MetS research.

Among the organs involved in metabolic regulation, adipose tissue plays a central role not only as an energy storage depot but also as an active endocrine organ that secretes adipokines capable of modulating insulin sensitivity, lipid metabolism, and inflammatory responses [15]. Expansion of visceral adipose tissue promotes macrophage infiltration and increased production of pro-inflammatory cytokines, thereby contributing to systemic inflammation and metabolic dysfunction [15,16]. Likewise, the liver plays a pivotal role through dysregulated gluconeogenesis, hepatic insulin resistance, and lipid accumulation, ultimately contributing to metabolic dysfunction-associated steatotic liver disease (MASLD), which is closely linked to MetS [17,18]. Skeletal muscle, the primary site of insulin-mediated glucose disposal, progressively develops insulin resistance and mitochondrial dysfunction, further aggravating metabolic inflexibility and impaired glucose utilization [19,20]. Pancreatic β-cells constitute a key component of metabolic regulation, as their dysfunction contributes to impaired glucose homeostasis and the development of type 2 diabetes. The activity of β-cells depends on their close interaction with the intra-islet microvasculature, composed of endothelial cells and pericytes. Beyond their role in maintaining islet blood supply, vascular cells regulate β-cell function through the secretion of growth factors and other signaling molecules that promote insulin gene expression, insulin secretion, and β-cell proliferation. This β-cell-vascular crosstalk represents an important mechanism involved in glucose regulation and pancreatic endocrine function [21].

Chronic low-grade inflammation has emerged as a fundamental mechanism underlying the development and progression of MetS [22,23]. Activation of innate immune pathways increased circulating inflammatory mediators, and oxidative stress, endothelial dysfunction, and altered mitochondrial bioenergetics collectively contribute to metabolic impairment [24]. Intracellular signaling pathways such as nuclear factor kappa B (NF-κB) and c-Jun N-terminal kinase (JNK) are persistently activated, disrupting insulin signaling and promoting metabolic abnormalities across multiple tissues [12,25,26,27]. Consequently, molecular pathways involved in energy sensing, inflammatory regulation, lipid metabolism, and oxidative stress have become key targets for understanding the biological complexity of MetS. Among these pathways, AMP-activated protein kinase (AMPK) and NF-κB have been extensively studied due to their central roles in regulating cellular energy balance, insulin sensitivity, mitochondrial metabolism, and chronic inflammatory responses associated with metabolic dysfunction [28,29].

Among the numerous factors implicated in metabolic regulation, vitamin D has gained considerable attention due to its pleiotropic biological functions extending far beyond calcium and bone homeostasis [30,31,32]. Through activation of the vitamin D receptor (VDR), which is widely expressed in adipocytes, hepatocytes, skeletal muscle, pancreatic β-cells, endothelial cells, and immune cells, vitamin D participates in the regulation of insulin sensitivity, lipid metabolism, inflammatory responses, oxidative stress, mitochondrial function, and immune modulation [33,34,35,36]. Vitamin D deficiency has been consistently associated with obesity, insulin resistance, hypertension, dyslipidemia, and other components of MetS, suggesting that inadequate vitamin D status may contribute to the development and progression of metabolic dysfunction [31,32,37]. Although the causal nature of these associations remains under investigation, accumulating experimental and clinical evidence indicates that vitamin D may influence several molecular pathways involved in the pathophysiology of MetS. However, whether vitamin D deficiency represents a causal driver of metabolic dysfunction or rather a consequence of obesity, inflammation, reduced physical activity, and altered metabolic status remains unresolved [38]. Furthermore, the extent to which vitamin D contributes to inter-organ communication through coordinated regulation of endocrine mediators, inflammatory pathways, and metabolic signaling networks has not been comprehensively integrated [39].

Previous reviews have primarily focused on isolated aspects of vitamin D biology, including associations with insulin resistance, inflammation, obesity, or individual metabolic outcomes [40,41]. In contrast, the present review proposes an integrative conceptual framework in which vitamin D is examined as a potential modulator of inter-organ communication involving adipose tissue, liver, skeletal muscle, pancreas, intestine, vascular endothelium, and immune cells [42].

Therefore, the aim of this review is to examine the role of vitamin D in the pathophysiology of MetS from a multisystem perspective. Specifically, this review summarizes current evidence regarding the molecular mechanisms through which vitamin D may influence inter-organ communication, insulin resistance, adipose tissue dysfunction, hepatic metabolism, skeletal muscle function, chronic inflammation, oxidative stress, and mitochondrial homeostasis, while critically distinguishing between direct experimental evidence, biological plausibility, observational associations, and potential clinical implications. This conceptual approach seeks to provide a more comprehensive understanding of vitamin D as a potential regulator of metabolic networks rather than as an isolated factor affecting individual components of MetS.

## 2. Pathophysiology of MetS as a Multisystem Disorder

MetS is a complex metabolic disorder characterized by the coexistence of central obesity, insulin resistance, hypertension, dyslipidemia, and impaired glucose metabolism, all of which markedly increase the risk of developing T2DM, cardiovascular disease (CVD), and premature mortality [1,2,3]. Rather than representing a simple clustering of independent metabolic abnormalities, MetS is currently recognized as a multisystem disorder involving coordinated dysfunction across several metabolically active organs and tissues [7,8,9,10,11].

Under physiological conditions, glucose and lipid homeostasis are tightly regulated through coordinated communication among the adipose tissue, skeletal muscle, liver, pancreas, intestine, kidneys, vascular endothelium, immune system, and central nervous system [19]. These organs interact through hormones, adipokines, cytokines, metabolites, and neuroendocrine signals to maintain metabolic balance [22]. In MetS, disruption of this inter-organ communication network results in insulin resistance, impaired lipid metabolism, chronic low-grade inflammation, oxidative stress, endothelial dysfunction, and progressive metabolic deterioration [22,24].

The development of MetS arises from complex interactions between genetic susceptibility, environmental exposures, and lifestyle-related factors [43]. Obesity, particularly excess visceral adiposity; physical inactivity; unhealthy dietary patterns; aging; sleep disturbances; and chronic psychosocial stress are among the principal contributors to its increasing global prevalence [44,45]. Expansion of visceral adipose tissue promotes excessive release of free fatty acids (FFAs), altered secretion of adipokines such as leptin and adiponectin, and increased production of pro-inflammatory cytokines, thereby establishing a chronic inflammatory state that exacerbates insulin resistance and metabolic dysfunction [46].

Adipose tissue represents one of the central drivers of MetS because of its endocrine and immunometabolic functions [16]. Dysfunctional adipose tissue recruits macrophages and other immune cells that produce inflammatory mediators, including tumor necrosis factor-alpha (TNF-α), interleukin-6 (IL-6), and monocyte chemoattractant protein-1 (MCP-1), which interfere with insulin signaling through activation of intracellular pathways such as NF-κB and c-Jun N-terminal kinase (JNK) [35,47,48]. These molecular alterations contribute to systemic inflammation, insulin resistance, and metabolic inflexibility.

The liver plays a pivotal role in MetS through increased hepatic gluconeogenesis, impaired lipid oxidation, de novo lipogenesis, and excessive triglyceride accumulation, leading to MASLD, a common hepatic manifestation of MetS [17,18]. Skeletal muscle, which accounts for most insulin-mediated glucose disposal, develops impaired insulin signaling, reduced glucose uptake, mitochondrial dysfunction, and decreased metabolic flexibility, thereby contributing to hyperglycemia and reduced energy utilization [49,50,51]. Pancreatic β-cells initially compensate for insulin resistance by increasing insulin secretion; however, prolonged exposure to glucotoxicity, lipotoxicity, oxidative stress, and inflammation progressively impairs β-cell function and may eventually lead to glucose intolerance and T2DM [31].

Increasing evidence also supports important contributions from other organ systems. The intestine influences metabolic regulation through nutrient absorption, secretion of incretin hormones such as glucagon-like peptide-1 (GLP-1), maintenance of intestinal barrier integrity, and interactions with the gut microbiota [52]. Dysbiosis and increased intestinal permeability promote systemic inflammation through translocation of microbial products and activation of innate immune responses [53]. The kidneys contribute by regulating sodium balance, blood pressure, and glucose reabsorption, whereas endothelial dysfunction promotes vascular stiffness, hypertension, and accelerated atherosclerosis [54]. Furthermore, the central nervous system integrates hormonal and nutritional signals that regulate appetite, energy expenditure, autonomic function, and neuroendocrine responses, all of which become dysregulated in MetS [55].

Mitochondrial dysfunction and oxidative stress have emerged as fundamental mechanisms linking these organ-specific alterations [56]. Excess nutrient availability increases reactive oxygen species (ROS) production, impairs mitochondrial bioenergetics, disrupts cellular signaling, and amplifies inflammatory pathways, thereby perpetuating insulin resistance and metabolic dysfunction across multiple tissues [56,57,58]. Consequently, MetS should be viewed as a systemic disorder resulting from the interaction of metabolic, inflammatory, endocrine, vascular, and mitochondrial abnormalities rather than isolated defects in individual organs.

Table 1 summarizes the principal clinical characteristics, diagnostic criteria, metabolic alterations, and major risk factors associated with MetS, complementarily.

**Figure 1:**
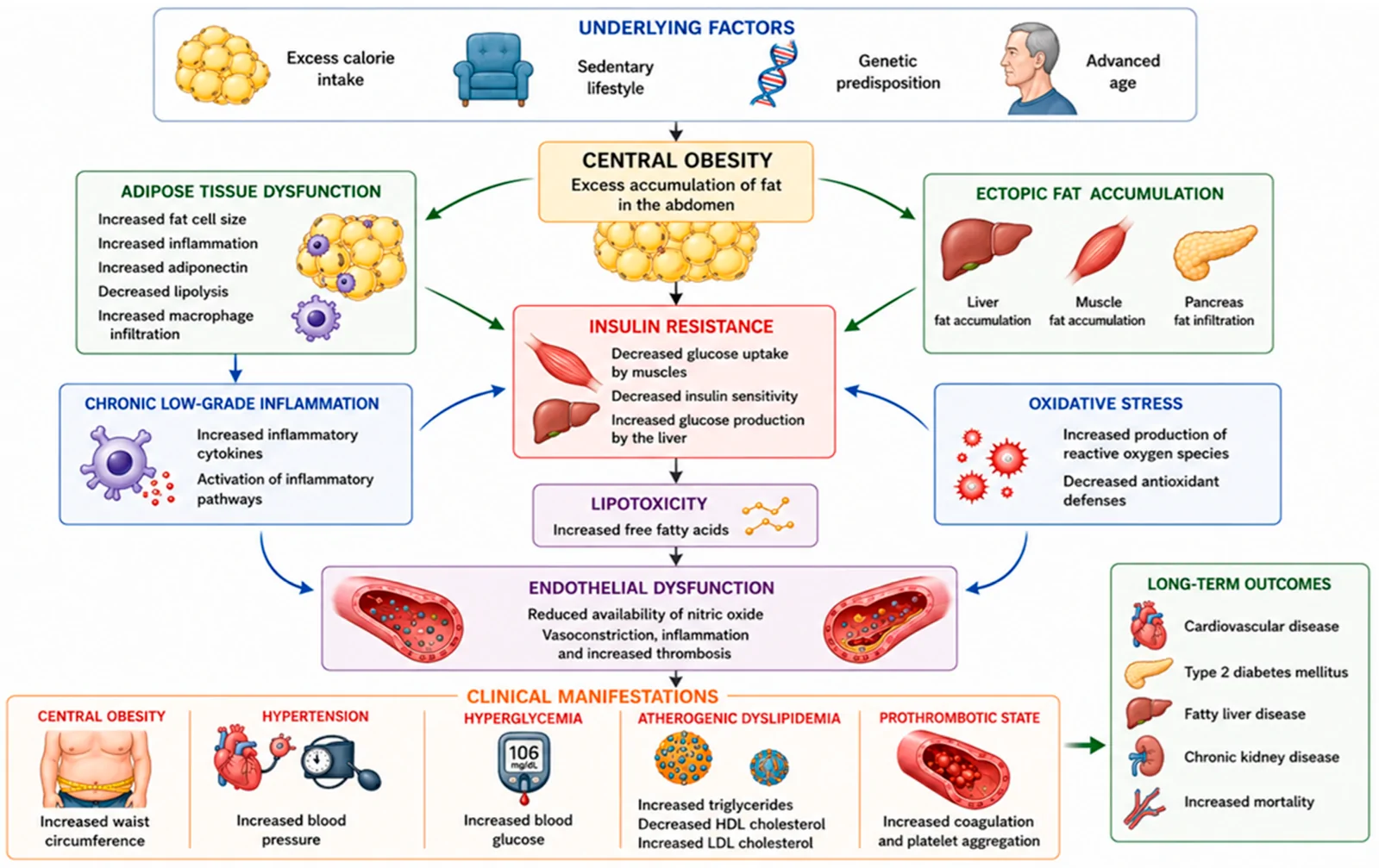
Pathophysiological background of MetS. Source: own elaboration.

Figure 1 illustrates the multisystem pathophysiology of MetS, highlighting the interactions among visceral adiposity, insulin resistance, chronic inflammation, endothelial dysfunction, mitochondrial impairment, oxidative stress, and altered inter-organ communication that collectively contribute to the onset and progression of the syndrome.

## 3. Vitamin D Metabolism

Vitamin D has emerged as an important regulator of metabolic health, extending its biological functions well beyond its classical role in maintaining calcium and phosphate homeostasis [66,67]. Increasing evidence indicates that inadequate vitamin D status may contribute to the onset and progression of MetS, a complex disorder characterized by the coexistence of abdominal obesity, insulin resistance, dyslipidemia, hypertension, and impaired glucose regulation [68]. Although the relationship between vitamin D deficiency and MetS remains under active investigation, epidemiological studies consistently report an inverse association between circulating 25-hydroxyvitamin D [25(OH)D] concentrations and the prevalence of MetS and its individual components [68,69,70,71].

Vitamin D is obtained through dietary intake as ergocalciferol (vitamin D_2_) or synthesized endogenously as cholecalciferol (vitamin D_3_) following exposure of the skin to ultraviolet B radiation [66,67]. Both forms are biologically inactive and require sequential hydroxylation in the liver and kidneys to generate 1,25-dihydroxyvitamin D (1,25(OH)_2_D), the hormonally active metabolite [72]. The biological actions of 1,25(OH)_2_D are mediated by the VDR, which is widely distributed in metabolically relevant tissues, including adipose tissue, skeletal muscle, pancreatic β-cells, the liver, the vascular endothelium, and immune cells [33,34,35,36]. Through this receptor, vitamin D regulates the transcription of numerous genes involved in glucose homeostasis, lipid metabolism, inflammation, and cellular energy balance [33,34,35,36].

One of the principal mechanisms linking vitamin D to MetS involves the regulation of insulin action [68]. Experimental and preclinical studies indicate that vitamin D induces autophagy, suppresses apoptosis of pancreatic β-cells, and prevents insulitis, thereby contributing to the preservation of β-cell function in diabetes [73]. In contrast, epidemiological studies and meta-analyses have consistently reported inverse associations between circulating 25(OH)D concentrations and the prevalence of MetS and its individual components, although these associations do not by themselves establish causality [74,75,76].

Chronic low-grade inflammation is another hallmark of MetS that appears to be influenced by vitamin D status [77]. Activation of VDR suppresses pro-inflammatory signaling pathways, particularly NF-κB, resulting in reduced production of inflammatory mediators such as TNF-α, interleukin-1β (IL-1β), and IL-6 [41,78,79,80]. Concurrently, vitamin D promotes the expression of anti-inflammatory cytokines, including interleukin-10 (IL-10), and favors macrophage polarization toward the anti-inflammatory M2 phenotype. These immunomodulatory effects may attenuate adipose tissue inflammation and contribute to improved metabolic function [41,81,82].

In addition to modulating inflammation, vitamin D has been implicated in the preservation of mitochondrial function and the control of oxidative stress. Experimental studies suggest that adequate vitamin D availability reduces reactive oxygen species (ROS) generation, enhances antioxidant defense systems, and preserves endothelial integrity [83,84]. These effects may help limit the progression of insulin resistance and vascular dysfunction, two major pathophysiological processes associated with MetS [55,56,83,84].

Although these molecular mechanisms provide strong biological plausibility, clinical evidence regarding vitamin D supplementation remains inconclusive. Differences in baseline vitamin D status, supplementation protocols, treatment duration, and participant characteristics have produced heterogeneous findings across randomized controlled trials [85,86]. Therefore, additional well-designed studies are needed to determine whether vitamin D supplementation can effectively prevent or improve the metabolic abnormalities associated with MetS [68,69,70,71].

**Figure 2:**
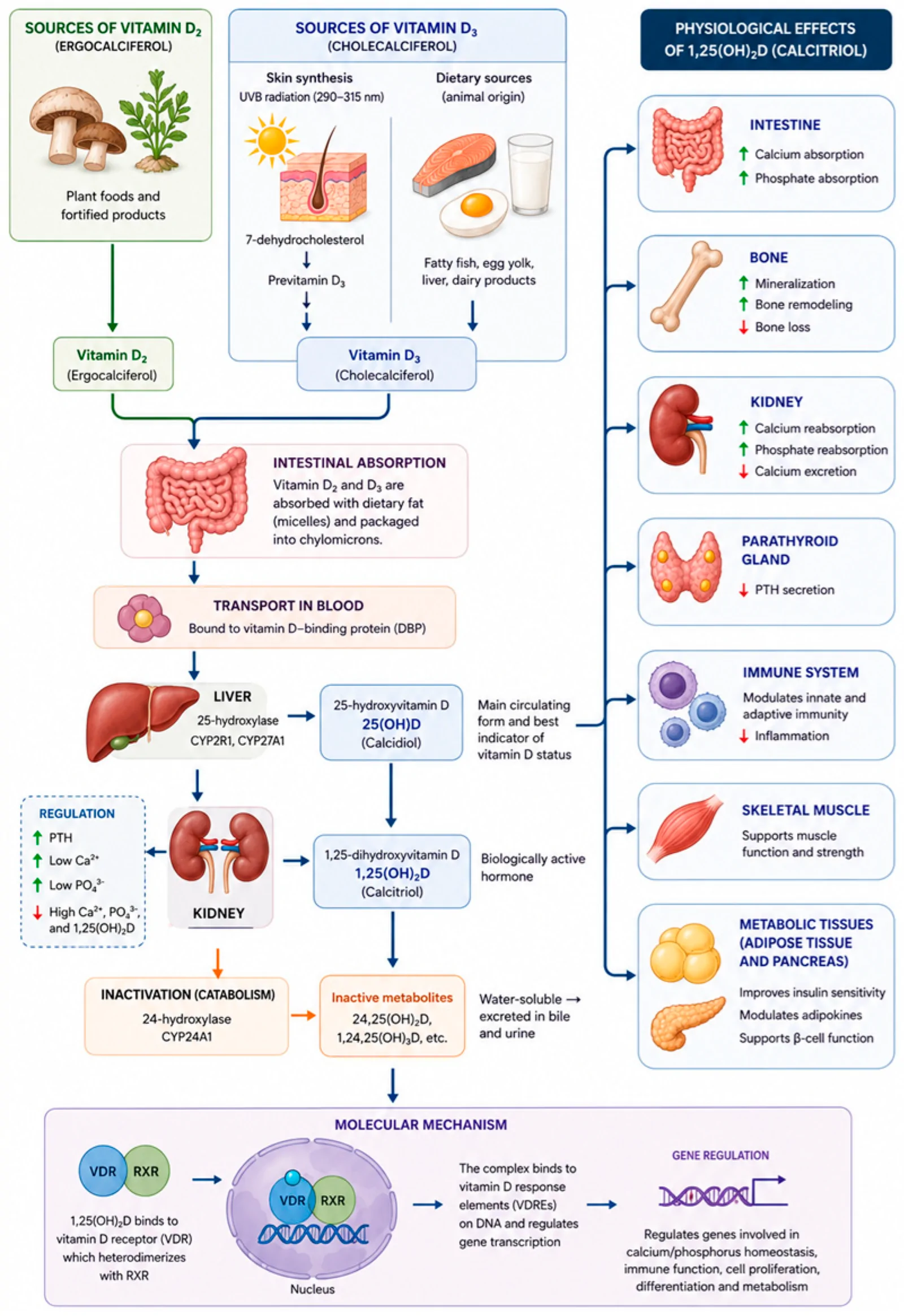
Vitamin metabolism. Source: adaptation of Fuentes-Barría et al. [31].

Figure 2 presents an overview of the proposed mechanisms through which vitamin D affects metabolic regulation.

### 3.1. Vitamin D Levels

Circulating vitamin D status is primarily assessed by measuring serum 25(OH)D, with results typically expressed in either nanograms per milliliter (ng/mL) or nanomoles per liter (nmol/L) [85]. Although these units are interchangeable, there is still no universally accepted standard for defining the thresholds that distinguish deficiency, insufficiency, and sufficiency. Consequently, the interpretation of serum 25(OH)D concentrations remains a subject of ongoing debate [85].

Among the most widely adopted reference values are those proposed by the U.S. Institute of Medicine (IOM), which classify severe vitamin D deficiency as serum concentrations between 10 and 12 ng/mL (25–30 nmol/L), deficiency as levels below 20 ng/mL (<50 nmol/L), and sufficient vitamin D status as concentrations exceeding 20 ng/mL (>50 nmol/L) [85,86].

Despite the widespread use of these criteria, no universal agreement exists regarding the optimal serum concentration of vitamin D. Different scientific organizations, including the International Osteoporosis Foundation and the American Geriatrics Society, recommend alternative cutoff values based on their interpretation of the available evidence [85,86]. These inconsistencies arise from several factors, including differences in laboratory methods used to quantify 25(OH)D, variability in study populations, distinct clinical endpoints related to skeletal health, and the influence of genetic, environmental, and lifestyle factors on vitamin D metabolism [87,88]. Importantly, many of the differences among recommendations issued by professional organizations reflect distinct clinical objectives and varying interpretations of the available evidence, rather than fundamental disagreement regarding the biological role of vitamin D [86,88]. As a result, the most appropriate vitamin D concentration may vary according to the characteristics and clinical needs of specific populations [85,86]. For instance, higher circulating levels are often recommended for older adults and individuals with chronic diseases, whereas lower concentrations may be considered adequate for healthy adults without additional risk factors [88].

Vitamin D deficiency represents a major global public health concern because of its high prevalence across diverse populations [85,88]. Individuals residing at higher latitudes are particularly vulnerable due to reduced exposure to ultraviolet B (UVB) radiation, which limits endogenous vitamin D synthesis [89]. Likewise, people with darker skin pigmentation tend to produce lower amounts of vitamin D following sun exposure because increased melanin content decreases the penetration of UVB radiation into the skin [90,91,92]. Together, these factors contribute to lower circulating vitamin D concentrations and may increase the risk of adverse metabolic outcomes, including type 2 diabetes and other chronic diseases [93]. Therefore, geographic location, skin pigmentation, and sun exposure patterns should be considered when designing preventive and therapeutic strategies aimed at improving vitamin D status [90,91,92].

**Figure 3:**
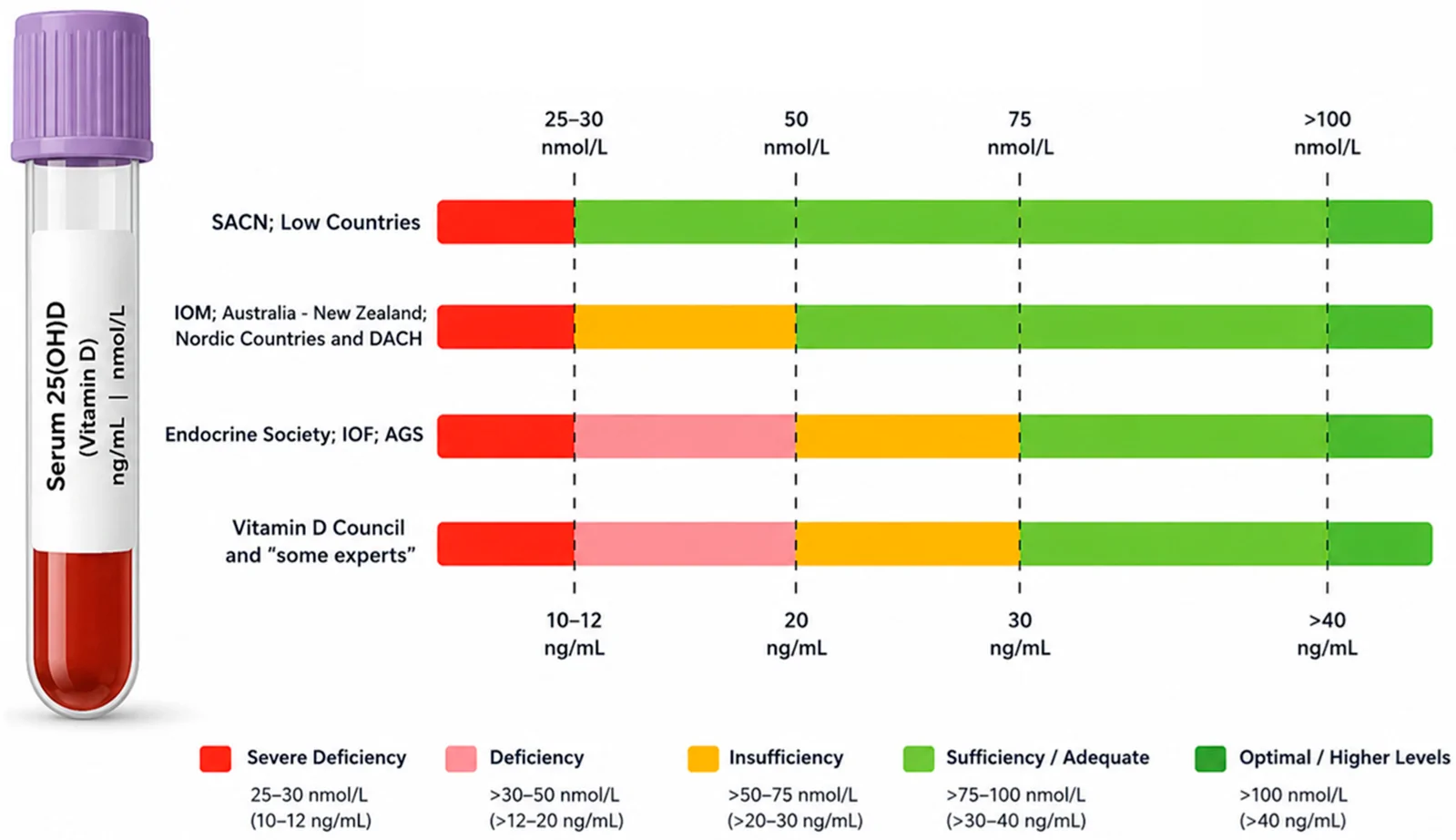
Comparison of 25(OH) serum level thresholds by organization. Source: adaptation of Herrera-Molina et al. [85]. SACN: Scientific Advisory Committee on Nutrition, IOM: Institute of Medicine, IOF: International Osteoporosis Foundation, AGS: American Geriatrics Society, DACH: Germany, Austria, and Switzerland.

Figure 3 provides a comparative overview of the serum 25(OH)D concentration ranges most used to classify vitamin D according to recommendations from major scientific organizations [85]. The figure illustrates the variability in the thresholds proposed to define severe deficiency, deficiency, insufficiency, sufficiency, and higher or optimal vitamin D levels, highlighting the absence of a universally accepted consensus. Serum 25(OH)D concentrations are presented in both nanograms per milliliter (ng/mL) and nanomoles per liter (nmol/L), the two standard units used in clinical practice and research [85]. While all organizations recognize severe deficiency at concentrations around 25–30 nmol/L (10–12 ng/mL), important differences emerge regarding the serum levels considered sufficient or optimal [85]. By visually comparing these recommendations, the figure emphasizes the heterogeneity among current clinical guidelines and illustrates how differences in interpretation of the available evidence have contributed to the lack of standardized cutoff values for serum vitamin D status.

### 3.2. Recommendations for Vitamin D Intake and Considerations

Maintaining adequate vitamin D status is considered an important component of overall health because of its involvement in multiple physiological processes beyond skeletal homeostasis [85,86,88]. In addition to its established role in calcium and phosphorus metabolism, vitamin D has been implicated in glucose regulation, insulin sensitivity, adipose tissue function, inflammatory responses, and cardiovascular health [66,67]. These biological effects have generated increasing interest in the potential contribution of vitamin D to the prevention and management of MetS and its individual components [68,69,70]. Consequently, ensuring an adequate vitamin D intake is particularly relevant not only for the general population but also for individuals at increased risk of vitamin D deficiency or metabolic disorders [85,86,88].

Current dietary recommendations vary according to age and physiological requirements. The Institute of Medicine recommends a daily intake of 10 μg (400 IU) during the first year of life; 15 μg (600 IU) for children, adolescents, and adults up to 70 years of age; and 20 μg (800 IU) for adults older than 70 years to compensate for the age-related decline in cutaneous vitamin D synthesis and reduced intestinal calcium absorption [86,94]. These recommendations are intended to maintain adequate serum 25(OH)D concentrations in healthy individuals and reduce the risk of deficiency.

Certain populations require special consideration because they are more likely to present low circulating vitamin D concentrations. Individuals with obesity, MetS, type 2 diabetes, chronic kidney disease, liver disorders, gastrointestinal malabsorption syndromes, or limited sun exposure frequently exhibit reduced vitamin D status [30,31,32,86,94]. In obesity, vitamin D is believed to become sequestered within adipose tissue, decreasing its bioavailability, whereas chronic inflammation and metabolic dysregulation may further impair vitamin D metabolism [95,96]. As a result, several clinical guidelines suggest that these individuals may require higher vitamin D supplementation than the general population to achieve equivalent serum 25(OH)D concentrations [85,86].

For adults at increased risk of deficiency, supplementation ranging from 1000 to 2000 IU/day is commonly recommended, with dosage adjustments based on baseline serum 25(OH)D concentrations, body composition, and clinical characteristics. In selected cases of confirmed deficiency or impaired vitamin D metabolism, higher doses may be prescribed under medical supervision, accompanied by periodic monitoring of serum 25(OH)D and calcium concentrations to ensure both efficacy and safety [86,94].

Environmental and geographic factors also play a major role in determining vitamin D status. Individuals living at higher latitudes or in regions with prolonged winters are exposed to lower levels of ultraviolet B (UVB) radiation, substantially reducing endogenous vitamin D synthesis [90,91,92]. Similar limitations may occur in populations with limited outdoor activity, extensive clothing coverage, or occupations that restrict sunlight exposure [90,91,92]. In these settings, public health strategies such as vitamin D food fortification and seasonal supplementation have proven effective in reducing the prevalence of hypovitaminosis D and may contribute to lowering the burden of metabolic disorders associated with vitamin D deficiency [86,94].

Although vitamin D supplementation is generally considered safe when administered within recommended limits, excessive intake can result in adverse effects. The tolerable upper intake level established for healthy adults is 4000 IU/day, while prolonged consumption of doses exceeding this level without medical supervision may increase the risk of hypercalcemia, hypercalciuria, nephrolithiasis, and soft tissue calcification [94]. Evidence of increased adverse events has been reported primarily in randomized trials employing annual bolus doses of 300,000–500,000 IU in older adults [97,98]. Evidence of increased adverse events has been reported primarily in randomized trials employing annual bolus doses of 300,000 IU in older adults. In contrast, other loading regimens using the same cumulative dose (300,000 IU) administered over 5–10 weeks have effectively corrected vitamin D deficiency without inducing 25(OH)D overload or increasing the risk of hypercalcemia, hypercalciuria, or changes in bone resorption markers, demonstrating a safety profile comparable to that of low-dose maintenance therapy [99]. For this reason, individualized supplementation based on serum 25(OH)D measurements remain preferable to empirical high-dose administration [86,94].

**Figure 4:**
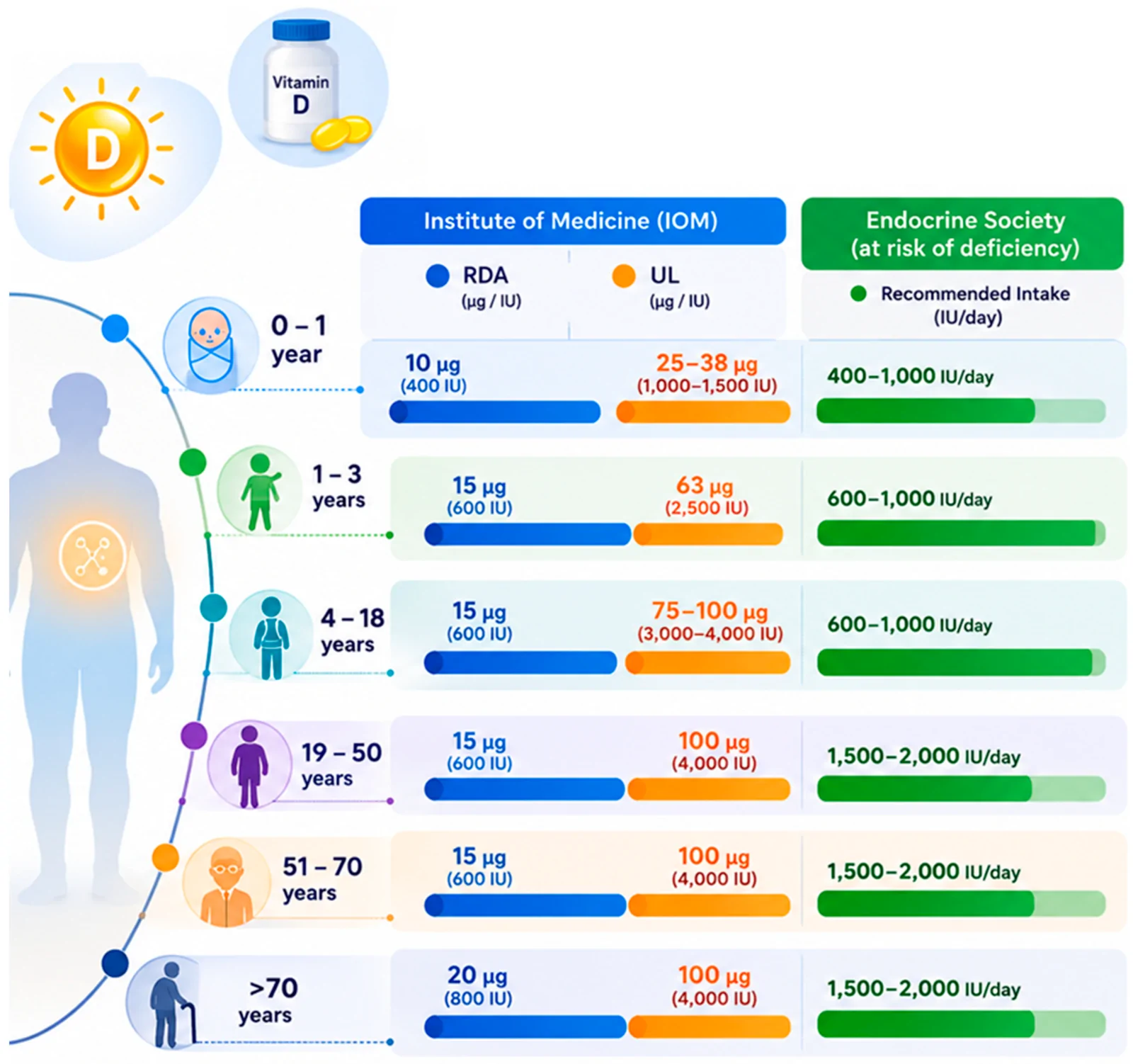
Recommended vitamin D intake by age according to the Institute of Medicine and Endocrine Society. Source: adaptation of Demay et al. [86], RDA: Recommended Dietary Allowance, UL: tolerable upper intake level, IU: International Units, μg: microgram.

Figure 4 summarizes the current recommendations for vitamin D intake according to age and clinical characteristics, with particular emphasis on populations at increased risk of MetS and vitamin D deficiency. The table also compares recommendations from major international organizations, illustrating how suggested supplementation strategies differ depending on the target population. While the Institute of Medicine provides recommendations for the general population, organizations such as the Endocrine Society, the International Osteoporosis Foundation, and the American Geriatrics Society advocate higher vitamin D intake for individuals with obesity, diabetes, osteoporosis, older age, or other chronic conditions that predispose to vitamin D deficiency. These differences reflect the growing recognition that vitamin D requirements should be individualized according to metabolic health, underlying disease, environmental exposure, and life stage rather than applying a single recommendation to all populations.

### 3.3. Vitamin D as a Molecular Modulator of MetS

Vitamin D has emerged as one of the most extensively investigated micronutrients in metabolic research because of its broad regulatory effects extending beyond calcium and bone homeostasis [100]. The biological actions of vitamin D are primarily mediated through activation of the VDR, a ligand-activated transcription factor expressed in adipocytes, skeletal muscle fibers, hepatocytes, pancreatic β-cells, vascular endothelial cells, macrophages, and intestinal epithelial cells [33,34,35,36]. Consequently, vitamin D influences multiple metabolic processes involved in the pathogenesis of MetS [101].

Experimental evidence suggests that vitamin D may influence several molecular pathways involved in cellular metabolism, inflammation, and stress responses; however, the available evidence is largely derived from specific experimental models and should not be directly extrapolated to adults with MetS [102,103,104]. In renal tubular cells, vitamin D signaling has been associated with modulation of pathways related to cellular stress responses, whereas studies in Kaposi sarcoma cells have provided mechanistic insights into vitamin D-mediated regulation of inflammatory and survival pathways [102,103]. Evidence from adipose tissue remodeling models further suggests potential effects of vitamin D signaling on lipid-related processes [104]. Nevertheless, these findings represent biological plausibility and provide mechanistic hypotheses rather than direct evidence of organ-specific regulation of AMPK, mTOR, NF-κB, Nrf2, mitochondrial function, insulin sensitivity, or metabolic control in patients with MetS. Further studies in relevant human metabolic tissues are required to clarify the contribution of vitamin D-mediated signaling pathways to MetS pathophysiology.

Within adipose tissue, vitamin D regulates adipocyte differentiation, suppresses macrophage infiltration, and reduces the production of pro-inflammatory cytokines, including TNF-α, IL-6, IL-1β, IL-8, and MCP-1 [105]. In addition, it promotes adiponectin secretion through modulation of peroxisome proliferator-activated receptor gamma (PPAR-γ) signaling, thereby contributing to the maintenance of adipose tissue homeostasis [105,106,107]. Vitamin D is associated with improved skeletal muscle structure and function, potentially through modulation of protein synthesis, mitochondrial metabolism, and energy production, thereby contributing to muscle strength and performance [108]. Recent experimental evidence further suggests that activation of hepatic VDR may attenuate the progression of MASLD. Specifically, activation of the VDR/HNF-4α/MTTP/ApoB signaling pathway has been shown to reduce hepatic lipid accumulation, enhance fatty acid β-oxidation, and improve insulin resistance, thereby supporting hepatic lipid homeostasis and identifying VDR-mediated signaling as a potential therapeutic target for MASLD [109]. Furthermore, vitamin D contributes to maintenance of intestinal barrier integrity and modulation of gut microbial composition, thereby reducing metabolic endotoxemia and systemic inflammation [110].

Despite strong mechanistic evidence, clinical findings remain inconsistent. Observational studies consistently report inverse associations between serum 25(OH)D concentrations and the prevalence of MetS, obesity, insulin resistance, and MASLD [111,112]. However, randomized controlled trials have demonstrated heterogeneous results regarding the effectiveness of vitamin D supplementation in improving metabolic outcomes [113,114]. These discrepancies likely reflect differences in baseline vitamin D status, obesity severity, supplementation dose, treatment duration, ethnicity, genetic polymorphisms affecting VDR signaling, concomitant lifestyle interventions, and study design [85,86]. In particular, individuals with severe vitamin D deficiency at baseline appear more likely to benefit from supplementation than vitamin D-replete participants, whereas obesity may reduce treatment responsiveness through volumetric dilution and sequestration of vitamin D in adipose tissue. In addition, differences in supplementation regimens (daily versus intermittent dosing, dose magnitude, and treatment duration), genetic variability influencing vitamin D metabolism and VDR signaling, and concurrent lifestyle interventions such as dietary modification and physical activity may substantially influence clinical responses. Collectively, these sources of heterogeneity should be considered when interpreting the existing evidence, as they may partly explain the inconsistent findings reported across randomized clinical trials [85,86,113,114].

Current evidence therefore suggests that vitamin D supplementation should not be regarded as an isolated therapeutic intervention but rather as one component of an integrated strategy combining healthy nutrition, regular physical activity, weight management, and optimization of metabolic health [115]. Individuals presenting with vitamin D deficiency may derive the greatest metabolic benefit, whereas supplementation in vitamin D-replete individuals appears to produce more modest effects [115].

### 3.4. Vitamin D and the Modulation of Inter-Organ Communication

The recognition that MetS results from disrupted communication among metabolically active organs has generated increasing interest in interventions capable of restoring inter-organ crosstalk [19]. Skeletal muscle, adipose tissue, liver, intestine, vascular endothelium, and immune cells participate in a complex network of endocrine, paracrine, and metabolic interactions that contribute to the regulation of systemic glucose and lipid metabolism, inflammatory responses, mitochondrial function, and energy homeostasis [42]. Although disruption of this inter-organ communication network is increasingly recognized as a key feature of MetS, the extent to which vitamin D directly modulates these interactions remains incompletely understood [19].

Vitamin D has been proposed as a potential regulator of metabolic communication due to the widespread expression of the VDR across metabolically active tissues [116]. Experimental studies suggest that VDR activation may influence the production of adipokines, myokines, hepatokines, cytokines, and intestinal mediators; however, most available evidence derives from specific cellular models and does not demonstrate direct causal communication between organs [117,118].

Within adipose tissue, vitamin D has been associated with reduced macrophage infiltration and modulation of inflammatory cytokine production, including TNF-α and IL-6, while some studies suggest potential effects on adiponectin secretion and insulin sensitivity [41,78,79,80]. These findings support the possible role of vitamin D in improving adipose tissue metabolic function and its interaction with peripheral organs, although direct evidence of restored inter-organ communication remains limited [119].

In skeletal muscle, vitamin D signaling has been linked to mitochondrial function, glucose metabolism, and muscle performance, potentially involving pathways related to AMPK and peroxisome proliferator-activated receptor gamma coactivator-1 alpha (PGC-1α). However, further evidence is required to establish whether these effects translate into enhanced muscle-derived endocrine regulation in individuals with MetS [120].

Vitamin D may also influence the gut–liver axis through effects on intestinal barrier integrity, microbial composition, and inflammatory signaling. Experimental studies suggest that vitamin D can modulate tight junction proteins, bacterial translocation, lipopolysaccharide (LPS)-related signaling, and Toll-like receptor 4 (TLR4)/NF-κB activation; nevertheless, these findings mainly represent tissue-specific mechanisms rather than demonstrated organ-to-organ communication [121,122]. Furthermore, VDR activity in adipose tissue models, including 3T3-L1 preadipocytes, has been associated with regulation of adipogenesis and lipogenesis, indicating that vitamin D effects on lipid metabolism may be highly context-dependent and influenced by the cellular environment. Therefore, these findings should not be directly interpreted as evidence of reduced hepatic lipogenesis or improved mitochondrial function in hepatocytes [123].

Collectively, current evidence suggests that vitamin D may participate in the regulation of metabolic communication networks through modulation of endocrine signaling, inflammatory pathways, oxidative stress, mitochondrial function, and nutrient-sensing mechanisms. However, direct evidence demonstrating that vitamin D restores inter-organ communication in humans with MetS remains insufficient, and further studies using integrated human metabolic models are needed to clarify these interactions [101,124].

From a systems biology perspective, these molecular mechanisms should not be interpreted as isolated events occurring within individual organs but rather as components of an integrated regulatory network that maintains whole-body metabolic homeostasis. Crosstalk among adipose tissue, skeletal muscle, liver, pancreas, intestine, vascular endothelium, and immune cells enables coordinated regulation of glucose and lipid metabolism, inflammatory responses, mitochondrial function, and energy balance. Consequently, disruption of one or more signaling pathways, including AMPK, mTOR, NF-κB, and PPAR-γ, may propagate metabolic dysfunction across multiple organs, thereby accelerating the progression of MetS. Conversely, interventions capable of simultaneously modulating these interconnected pathways, such as optimization of vitamin D status together with lifestyle modification, may contribute to restoring systemic metabolic homeostasis rather than improving isolated metabolic abnormalities.

## 4. Limitations of the Current Evidence

The understanding of the relationship between vitamin D and MetS has expanded considerably over the past decades. Nevertheless, important methodological and conceptual limitations continue to restrict the interpretation of available evidence and its translation into clinical practice and public health recommendations. Recognizing these limitations is crucial for improving future investigations and clarifying the therapeutic and preventive role of vitamin D in MetS.

A major source of inconsistency arises from the considerable heterogeneity among published studies. Differences in the diagnostic criteria used to define MetS, characteristics of the study populations, baseline vitamin D status, supplementation protocols, intervention duration, and measured outcomes reduce comparability across studies and hinder the development of robust conclusions regarding the effectiveness of vitamin D supplementation in individuals with MetS [74,76,101,115,125,126].

Beyond its classical role in calcium homeostasis, vitamin D has emerged as an important regulator of epigenetic mechanisms involved in metabolic health. Through its interaction with the VDR, it influences DNA methylation, histone acetylation, and transcriptional regulation of genes associated with chronic inflammation, lipid metabolism, adipogenesis, and insulin signaling [127]. These epigenetic effects may contribute to the modulation of pathways involved in the development and progression of MetS. For example, vitamin D has been reported to reduce methylation of the *IRS1* promoter, thereby enhancing insulin signaling in peripheral tissues [77,78,79,80,128]. Furthermore, VDR-mediated regulation of histone acetylation influences adipocyte differentiation and immune function, while genetic polymorphisms in enzymes involved in vitamin D metabolism, such as *CYP27B1*, may modify individual responses to supplementation through interactions with these epigenetic processes [129,130,131].

Increasing attention has also been directed toward the interaction between vitamin D and the intestinal microbiota as a potential mechanism underlying metabolic regulation. Vitamin D appears to promote the proliferation of beneficial microorganisms, including *Bifidobacterium* and *Lactobacillus*, while suppressing pathogenic bacterial populations [132]. Additionally, it contributes to maintaining intestinal barrier integrity by regulating tight junction proteins, including claudins and occluding, thereby limiting bacterial lipopolysaccharide translocation and reducing chronic low-grade inflammation [133]. Emerging evidence suggests that vitamin D-induced alterations in gut microbial composition enhance the production of short-chain fatty acids, which may improve insulin sensitivity, adipose tissue function, and systemic inflammatory status—central components of MetS [134].

The mTOR pathway represents another important biological mechanism linking vitamin D to metabolic regulation. As a master regulator of cellular growth, nutrient sensing, and energy metabolism, excessive activation of mTORC1 has been associated with obesity, adipose tissue dysfunction, insulin resistance, and chronic inflammation. Experimental evidence indicates that vitamin D may attenuate excessive mTOR signaling, thereby reducing inflammatory responses and improving metabolic homeostasis, suggesting a potential role in mitigating key pathophysiological features of MetS [135,136,137,138].

Another limitation concerns the assessment of vitamin D status. Current laboratory methods for measuring circulating 25(OH)D, including high-performance liquid chromatography (HPLC) and immunoassays, differ in sensitivity and analytical performance. This lack of methodological standardization complicates comparisons among studies and contributes to uncertainty regarding the optimal thresholds used to define vitamin D deficiency, insufficiency, sufficiency, and toxicity [85,139].

The interpretation of observational and interventional studies examining the relationship between vitamin D status is complicated by the presence of multiple potential confounding factors. Lifestyle and biological factors, including physical activity, dietary patterns, obesity, body fat distribution, sun exposure, and genetic variability, may influence circulating vitamin D concentrations while also being independently associated with metabolic risk [140,141,142,143]. Therefore, the independent contribution of vitamin D status or supplementation to MetS-related outcomes remains difficult to establish. Moreover, environmental conditions, socioeconomic characteristics, physiological states, and individual genetic background may modify vitamin D metabolism, bioavailability, and biological responses, although the magnitude and clinical relevance of these interactions remain incompletely understood [144,145]. Importantly, current evidence should be interpreted considering the possibility of residual confounding, as low vitamin D concentrations may represent not only a potential biological factor involved in metabolic dysfunction but also a marker of adverse lifestyle patterns, adiposity, chronic disease burden, or other metabolic risk conditions. Most available clinical trials have relatively short follow-up periods, limiting the evaluation of the long-term effects of vitamin D supplementation on the prevention or progression of MetS. Additional variability is introduced by differences in vitamin D formulations, doses, administration schedules, and the use of vitamin D_2_ versus vitamin D_3_ [86,146].

Another important gap in the literature concerns the interaction between vitamin D supplementation and other evidence-based interventions for MetS, including structured exercise programs, dietary modification, weight-loss strategies, and pharmacological therapies [74,76,101,115,125,126]. Evaluating these combined approaches may provide a more comprehensive understanding of the contribution of vitamin D to cardiometabolic health and help identify patient subgroups most likely to benefit from supplementation [126].

Current clinical guidelines do not recommend routine vitamin D supplementation specifically for the prevention or treatment of MetS in individuals without documented deficiency [86]. Although meta-analyses have consistently reported an inverse association between circulating vitamin D concentrations and the risk of MetS, the evidence regarding the benefits of vitamin D supplementation remains inconsistent, with no clear or clinically meaningful improvements observed across the components of MetS [74,76,101,115,125,126]. Consequently, current recommendations remain focused on correcting vitamin D deficiency or treating individuals at increased risk, such as older adults, pregnant women, or populations with limited sun exposure or inadequate dietary intake [86].

Overcoming these methodological limitations will be essential for future research to better define the contribution of vitamin D to the prevention and management of MetS. Well-designed randomized controlled trials with standardized diagnostic criteria, optimized supplementation protocols, longer follow-up periods, and comprehensive assessment of confounding variables will be necessary to establish whether vitamin D supplementation can provide meaningful clinical benefits for individuals with MetS.

Table 2 summarizes the current body of evidence evaluating the relationship between vitamin D and MetS, including studies investigating the effects of vitamin D supplementation on the individual components of MetS, insulin resistance, obesity-related outcomes, lipid metabolism, blood pressure regulation, systemic inflammation, and long-term cardiometabolic health.

## 5. Clinical Implications and Future Research Directions

MetS is a multifactorial disorder characterized by the coexistence of central obesity, insulin resistance, dyslipidemia, hypertension, chronic low-grade inflammation, and MASLD, all of which substantially increase the risk of T2DM, cardiovascular disease, and premature mortality. Rather than representing isolated metabolic abnormalities, these conditions arise from complex interactions among multiple organs mediated through endocrine, inflammatory, neural, and metabolic signaling pathways. Consequently, understanding the molecular mechanisms governing inter-organ communication provides an important foundation for developing more effective preventive and therapeutic strategies.

Current management of MetS relies primarily on comprehensive lifestyle modification, including dietary optimization, regular physical activity, weight reduction, adequate sleep, and management of cardiovascular risk factors. These interventions exert their beneficial effects through coordinated modulation of intracellular signaling pathways involved in energy sensing, mitochondrial function, oxidative stress, inflammation, and nutrient metabolism. In particular, activation of AMPK, suppression of excessive mechanistic mTOR signaling, attenuation of NF-κB-mediated inflammation, enhancement of nuclear factor erythroid 2-related factor 2 (Nrf2)-dependent antioxidant responses, and regulation of PPAR-γ collectively contribute to improved insulin sensitivity, metabolic flexibility, and systemic metabolic homeostasis.

Accumulating evidence suggests that vitamin D may complement these lifestyle interventions through activation of the VDR, which is expressed in the adipose tissue, skeletal muscle, liver, pancreatic islets, vascular endothelium, immune cells, and intestinal epithelial cells. Experimental studies indicate that vitamin D modulates inflammatory signaling, mitochondrial function, oxidative stress, adipocyte biology, endothelial function, gut barrier integrity, and inter-organ communication. Through these mechanisms, vitamin D has the potential to influence several pathophysiological components of MetS simultaneously rather than targeting a single metabolic abnormality.

Nevertheless, despite robust mechanistic evidence, the clinical effectiveness of vitamin D supplementation remains uncertain. Observational studies consistently demonstrate inverse associations between serum 25(OH)D concentrations and the prevalence of MetS and its individual components. However, randomized controlled trials and meta-analyses have produced heterogeneous findings regarding improvements in insulin resistance, lipid profile, blood pressure, body composition, and inflammatory biomarkers following vitamin D supplementation. These inconsistencies likely reflect substantial differences in baseline vitamin D status, obesity severity, supplementation dose and duration, ethnicity, genetic polymorphisms affecting VDR signaling, concomitant lifestyle interventions, and study design.

These findings emphasize that vitamin D supplementation should not be considered a stand-alone treatment for MetS but rather an adjunctive strategy within comprehensive lifestyle-based management. Individuals with documented vitamin D deficiency, obesity, or increased cardiometabolic risk may represent the population most likely to benefit from vitamin D optimization, although additional high-quality clinical evidence is required to establish personalized supplementation strategies.

Future research should focus on identifying the biological mechanisms that determine individual responsiveness to vitamin D. Integrating genomics, epigenomics, transcriptomics, proteomics, metabolomics, microbiome profiling, and systems biology approaches may facilitate the identification of biomarkers capable of predicting treatment response and disease progression. Particular attention should be directed toward genetic polymorphisms affecting VDR function, vitamin D metabolism, inflammatory pathways, mitochondrial regulation, and nutrient-sensing networks.

Large, well-designed randomized controlled trials are also needed to determine the optimal dose, duration, and timing of vitamin D supplementation according to baseline vitamin D status, obesity severity, metabolic phenotype, and concomitant lifestyle interventions. Future studies should evaluate clinically meaningful outcomes, including insulin sensitivity, visceral adiposity, hepatic steatosis, endothelial function, cardiovascular events, and progression to T2DM, rather than relying exclusively on surrogate biochemical markers.

Ultimately, the future management of MetS will likely depend on precision medicine approaches integrating molecular profiling, lifestyle interventions, and targeted nutritional strategies. Within this framework, vitamin D should be considered one component of a broader systems-based approach aimed at restoring inter-organ communication, improving metabolic flexibility, reducing chronic inflammation, and preserving long-term cardiometabolic health. Continued investigation into the molecular mechanisms linking vitamin D signaling with metabolic regulation will be essential for translating experimental discoveries into effective clinical interventions.

## 6. Conclusions

MetS is a complex multisystem disorder characterized by disrupted molecular signaling and impaired communication among metabolically active organs. Alterations in key pathways, including AMPK, mTOR, NF-κB, and PPAR-γ, contribute to insulin resistance, chronic inflammation, oxidative stress, mitochondrial dysfunction, and metabolic imbalance.

Accumulating evidence suggests that vitamin D, through activation of the VDR, acts as a pleiotropic regulator of these interconnected pathways. Beyond its classical role in calcium homeostasis, vitamin D influences adipose tissue function, skeletal muscle metabolism, hepatic lipid metabolism, gut barrier integrity, and inter-organ communication, highlighting its potential contribution to maintaining metabolic homeostasis.

Despite strong mechanistic evidence, clinical studies evaluating vitamin D supplementation have reported inconsistent results. These discrepancies likely reflect differences in baseline vitamin D status, obesity severity, supplementation protocols, and population characteristics, suggesting that vitamin D should be considered an adjunct to comprehensive lifestyle interventions rather than a stand-alone therapy.

Overall, this review supports a systems biology perspective in which MetS is viewed as a disorder of disrupted molecular signaling and organ crosstalk. Integrating vitamin D optimization with healthy dietary patterns, regular physical activity, and personalized therapeutic strategies may represent a promising approach to improve metabolic health. Future well-designed clinical trials and multi-omics studies are needed to clarify the clinical role of vitamin D and identify individuals most likely to benefit from supplementation.

## Figures and Tables

**Table 1:** Principal clinical characteristics, diagnostic criteria, metabolic alterations, and major risk factors associated with MetS.

Component	Clinical Characteristics	Diagnostic Criteria	Key Metabolic Alterations	Major Risk Factors	References
Central obesity	↑ Visceral adiposity	↑ Waist circumference	Adipose dysfunction	Sedentary lifestyle, excess calories and genetics	[59,60,61,62].
Hyperglycemia	↑ Fasting glucose	≥100 mg/dL or treatment	Insulin resistance	Obesity, physical inactivity and family history	[59,60,61,62].
Hypertriglyceridemia	↑ Triglycerides	≥150 mg/dL or treatment	↑ VLDL production	Insulin resistance and obesity	[59,60,61,62].
Low HDL-C	↓ HDL-C	Men <40; women <50 mg/dL or treatment	↓ Reverse cholesterol transport	Smoking, obesity and physical inactivity	[59,60,61,62].
Hypertension	↑ Blood pressure	≥130/85 mmHg or treatment	Endothelial dysfunction	Obesity, aging and excess sodium intake	[59,60,61,62].
Inflammation	Low-grade inflammation	-	↑ TNF-α ↑ IL-6 ↑ NF-κB	Visceral obesity	[62,63].
Oxidative stress	↑ ROS	-	Mitochondrial dysfunction	Hyperglycemia and chronic inflammation	[62,64].
Metabolic inflexibility	Impaired substrate utilization	-	↓ Mitochondrial oxidation	Obesity and physical inactivity	[62,63,64,65].

HDL-C, high-density lipoprotein cholesterol; VLDL, very-low-density lipoprotein; ROS, reactive oxygen species; TNF-α, tumor necrosis factor-α; IL-6, interleukin-6; NF-κB, nuclear factor kappa B.

**Table 2:** Summary of current evidence on MetS.

Author	Purpose	Conclusion
Hajhashemy et al. [74].	Quantify the association between blood vitamin D levels and risk of MetS in adults.	This meta-analysis reported an inverse association between serum vitamin D concentrations and MetS risk, but prospective evidence remains insufficient to confirm causality.
Rouhani et al. [76].	To meta-analyze the relationship between circulating 25(OH)D and MetS in children in epidemiological studies.	Higher serum vitamin D concentrations were inversely associated with the risk of MetS in children in a dose–response manner.
Totonchi et al. [101].	Conducted a meta-analysis to clarify the exact association between the VDR polymorphisms and the risk of MetS.	The VDR BsmI (rs1544410) polymorphism may be a protective genetic factor against MetS.
Huang et al. [115].	This meta-analysis compared the metabolic effects of different vitamin D formulations in patients with obesity-associated MetS.	Vitamin D_3_ supplementation may improve metabolic outcomes in obesity-associated MetS, while vitamin D_2_ appears less effective.
AlAnouti et al. [125].	To summarize the available evidence of randomized controlled trials to establish the impact of Vitamin D supplementation on dyslipidemia among adult patients with MetS.	Vitamin D supplementation seems not to affect blood lipids in adults with MetS.
Tabrizi et al. [126].	To summarize the effect of vitamin D supplementation on endothelial activation among patients with MetS and related disorders.	Vitamin D supplementation improved von Willebrand factor levels but had no significant effects on other endothelial biomarkers in patients with MetS.

## Data Availability

No new data were created or analyzed in this study. Data sharing is not applicable to this article.
